# Retraction: Al-Behadili, F.J.M., et al. Cold Responses of the Mediterranean Fruit Fly *Ceratitis capitata* Wiedemann (Diptera: Tephritidae) in Blueberry. *Insects* 2020, *11*, 276

**DOI:** 10.3390/insects11060360

**Published:** 2020-06-09

**Authors:** Farhan J.M. Al-Behadili, Manjree Agarwal, Wei Xu, Yonglin Ren

**Affiliations:** 1 College of Science, Health, Engineering and Education, Murdoch University, Murdoch, WA 6150, Australia; f.al-behadili@murdoch.edu.au (F.J.M.A.-B.); m.agarwal@murdoch.edu.au (M.A.); 2 College of Agriculture, Misan University, Misan 62001, Iraq

The published article [1] has been retracted and removed at the request of Murdoch University owing to legal issues of confidentiality. The University was not aware that the article had been submitted for publication and did not agree to the publication of the paper in its current form. Thus, out of respect for the institution’s wishes, in agreement with the *Insects* Editorial Office and in agreement with the authors, the paper will be removed from the public record and marked as retracted. We apologize for any inconvenience caused by the removal of this article.

*Insects* is a member of the Committee on Publication Ethics (COPE) and strives to uphold the highest ethical standards. The article [1] is retracted and shall be marked accordingly.
